# When does sleep affect veridical and false memory consolidation? A meta-analysis

**DOI:** 10.3758/s13423-018-1528-4

**Published:** 2018-09-27

**Authors:** Chloe Rhianne Newbury, Padraic Monaghan

**Affiliations:** Lancaster University, Lancaster, UK

**Keywords:** Sleep, False memory, Memory consolidation, Meta-analysis

## Abstract

It is widely accepted that sleep aids in the encoding, consolidation, and retrieval processes involved in memory processing; however, the conditions under which sleep influences memory may be substantially constrained. In a meta-analysis, we examined the effects that sleep has on both veridical (accurate) and false memory consolidation, in studies using the Deese/Roediger–McDermott (DRM) paradigm for memory of thematically related words. The meta-analysis revealed that, whereas there was no overall effect of sleep on either accurate or false memories, the effect of sleep on overall memories was moderated by two constraints. First, sleep effects were influenced by the number of words within each themed word list, relating to differences in processing of the associative network of related words. Second, sleep effects were greater in recall than in recognition tests. Thus, whether sleep consolidation increased or decreased DRM veridical or false memory effects depended on the specific features of the memory task.

Sleep benefits both the encoding and retrieval processes involved in memory consolidation, improving both declarative and procedural memory relative to the same time spent awake (Rasch & Born, 2007, 2013; Stickgold, 2005; Walker & Stickgold, 2006). The active systems consolidation hypothesis (Diekelmann & Born, 2010; Marshall & Born, 2007; Rasch & Born, 2013) suggests that information and events that we are exposed to during wakefulness are encoded initially in the hippocampus and neocortical systems. Consolidation during sleep then leads to repeated reactivation of these encoded memory representations, leading to an integration of selective information in the neocortex, where the memory is established in the long-term store (Lewis & Durrant, 2011). Substantial evidence supports this theory: For example, declarative memory for word pairs has been found to be greater after a delay that includes a period of sleep than after a delay spent completely awake (Gais & Born, 2004; Plihal & Born, 1997; Wilson, Baran, Pace-Schott, Ivry, & Spencer, 2012).

Several studies have also tested the hypothesis that sleep not only affects the processing and consolidation of previously experienced material, but also impacts the formation of false memories. The Deese/Roediger–McDermott (DRM) paradigm (Roediger & McDermott, 1995) has been extensively used to test when unseen related information, termed *false memories*, is activated in memory. In this paradigm, participants are exposed to lists of semantically related words (e.*g*., *bed*, *dream*, *tired*, *snooze*, *yawn*, etc.) and are asked to recall or recognize words previously seen in the initial lists. Words are categorized as either those that had appeared in the initial lists (*old* words); words that did not appear in the lists but were closely related, known as *lure* words (e.g., *sleep* in the list above); or unseen, unrelated words (*new* words). Participants are more likely to recall, or identify as previously seen, lure words than new words, demonstrating the false memory effect (McDermott, 1996; Roediger & McDermott, 1995; Roediger, Watson, McDermott, & Gallo, 2001).

Whilst evidence for sleep’s effect on veridical memory performance has been widely replicated, the question of whether sleep has an effect on DRM false memories remains. Potential inconsistencies in results emerge between tests of recall, in which false memories seem to be enhanced by sleep (Diekelmann, Born, & Wagner, 2010; Payne et al., 2009), and tests of recognition, in which sleep has been observed to enhance false memories, to have no effect, or even to reduce false memories (Diekelmann, Landolt, Lahl, Born, & Wagner, 2008; Fenn, Gallo, Margoliash, Roediger, & Nusbaum, 2009; Monaghan, Shaw, Ashworth-Lord, & Newbury, 2017). The activation/monitoring framework (Collins & Loftus, 1975) provides one possible explanation for the differences found between DRM tests of recall and recognition. The framework proposes that during tests of recognition, monitoring cues are activated when the words are presented to participants, allowing for the suppression of related but unseen words (Watson, McDermott, & Balota, 2004). During tests of recall, these monitoring cues are not available, and so a greater number of associated words are activated. This leads to greater false memory in tests of recall than of recognition. Sleep has been found to improve source-monitoring abilities (Johnson, Hashtroudi, & Lindsay, 1993), and therefore improves the ability to reject unseen related items during tests of recognition to a greater extent than during recall.

This difference in memory performance between tests of recall and recognition has been suggested in a meta-analysis of only a small number of studies that were published at the time (Chatburn, Lushington, & Kohler, 2014). A small, nonsignificant effect of sleep on false recognition was found, whereas false recall led to a large significant increase in false memory. However, this study examined the overall effect of only four studies in total, two studies on false recognition, and two on false recall. Therefore, the reliability of the effect of sleep on both false recall and recognition is still under review. To address this, the present meta-analysis included a larger sample of DRM studies, with five individual experiments examining the effect of sleep on false recall, and eight experiments investigating false recognition. This allowed for a more detailed exploration as to the effects of the two methods of testing, and also a greater understanding as to whether the effect of sleep on false memories does in fact reliably differ between tests of recall and recognition.

The larger number of experiments analyzed in this meta-analysis also permitted investigation of other potential moderator variables that might contribute to the effect of sleep on memory consolidation and the production of false memories within the DRM paradigm. In particular, we could determine whether the number of words in each list and the total number of lists that participants were required to remember influence the false memory effect. Using the DRM paradigm in a standard memory test (so, not testing the effect of sleep), Robinson and Roediger (1997) investigated the effect of varying list lengths on false recall and recognition. They found that increasing list length led to increases in both false recall and false recognition. Robinson and Roediger suggested that a larger number of words in each list increases the opportunity for participants to develop associations between the words, and therefore primes a larger number of unseen, related words during testing.

A possible explanation for the generation of false memories in DRM tests is spreading activation (Collins & Loftus, 1975). Word lists that participants are exposed to can activate unseen words that are similar in meaning to previously seen words. The associative activation theory (AAT) of false memories suggests that these lure words are activated due to their similarity or association with the seen words (Howe, Wimmer, Gagnon, & Plumpton, 2009; Roediger et al., 2001). Lists with greater strength of semantic association with the critical lure elicit increased false memories than do lists with weaker associations, due to spreading activation among associates within semantic memory (Gallo & Roediger, 2002). Alternatively, fuzzy trace theory (Payne et al., 2009) argues that false memories are a consequence of participants determining the gist or general theme of a list, and then activating all words related to that general meaning (Howe & Wilkinson, 2011). The mechanism of gist generation could again be due to spreading activation, with the theme generated as a consequence of interactive activation among associated words.

If sleep leads to greater spread of activation of previously seen word lists due to AAT or FTT (as was proposed by Cai et al., 2009; Sio, Monaghan, & Ormerod, 2013), then we should expect to see an increase in false recall and recognition of lure words after sleep in comparison to wakefulness. These theories raise predictions about the extent to which manipulating the density of inter-relations between words in a thematically related list affects the role of sleep in consolidation. A longer list of related words is more densely interconnected (Robinson & Roediger, 1997), and so spreading activation will occur to a greater extent for both sleep and wake groups equally. Thus, the benefit of sleep-related spreading activation is less likely to be detected than in a shorter list of related words, where the lure word’s concept receives only weak activation from a small set of related words within semantic associative memory (see Shaw & Monaghan, 2017, for a similar argument related to hemispheric processing). Hence, list length may be a critical factor in determining whether veridical and false memories are promoted by sleep. Indeed, previous research suggests that sleep is more beneficial when task difficulty increases, for both motor skills tasks (Stickgold & Walker, 2004), and problem solving tasks (Sio et al., 2013). If increasing the number of words in each list leads to closer associations and so easier access to semantically similar lure words, then we would expect sleep to increase false memories for studies with fewer words in each list, as activation of the lure word, or the theme, is more difficult to accomplish, so greater spreading activation is required across semantic networks.

Similar principles could also be expected to apply to the number of different lists that participants are exposed to. For instance, source monitoring is likely to become more difficult with larger numbers of lists, thereby increasing the likelihood of false memories, and decreasing veridical memory. Spreading activation across a large number of distinct thematic lists may also mitigate the potential effect of sleep on the generation of false memories.

The modality of presentation of word lists has also been found to affect the formation of DRM false memories. Previous research suggests differences in performance following visual as compared to auditory presentation of word lists. For both tests of recall and recognition, research indicates a significant reduction in the false memory effect when words are presented visually (Kellogg, 2001; Smith & Hunt, 1998). However, this difference in the effect of modality on false memory performance has been found to only be significant in those participants with higher working memory capacity (Smith & Engle, 2011). This difference in performance between visually and auditorily presented word lists was not found for veridical memory (Smith & Engle, 2011; Smith & Hunt, 1998). It is therefore of interest to assess modality as a potential moderator in the present meta-analysis.

The emotionality of to-be-remembered word lists may also influence the size of the effect of sleep on both accurate and false memories. Research has indicated an increase in overall memory performance for information with positive or negative emotional valence (Adelmann & Estes, 2013; Kensinger & Corkin, 2003). Furthermore, emotionality of word lists has also been found to increase false recognition in DRM tests (Howe, Candel, Otgaar, Malone, & Wimmer, 2010; Sharkawy, Groth, Vetter, Beraldi, & Fast, 2008), however possible differences arise between lists of negative and positive valence, with an increase in false recognition of negative word lists, and a decrease in false recognition of positive lists, relative to lists rated as neutral (Brainerd, Stein, Silveira, Rohenkohl & Reyna, 2008). The effect of emotionality on false recall is less clear. Bauer, Olheiser, Altarriba, and Landi (2009) suggest an increase in false recall for emotional word lists, whereas Howe et al. (2010) suggest a reduction in false recall for emotional as compared to neutral word lists.

Sleep has been suggested to further enhance this bias for the consolidation of emotional information, with studies indicating a role of rapid eye movement (REM) sleep specifically in the processing of emotional memories (Carr & Nielsen, 2015; Goldstein & Walker, 2014). Cai et al. (2009), and Carr and Nielsen (2015) suggested that REM sleep increases spreading activation, and hence that performance differences may be evident between emotional and neutral word lists after sleep. We thus tested emotionality of word lists as a potential moderator in the present meta-analysis, to assess whether emotionality leads to enhanced effects of sleep as compared to being awake on both veridical and false memories.

This larger set of studies included in a meta-analysis of DRM sleep-related effects means that we could also assess daytime nap versus overnight sleep effects on veridical and false memories. If sleep leads to greater spreading activation to semantic associates (Collins & Loftus, 1975), then we would expect an increase in time spent asleep to result in improved veridical performance as well as enhanced false memories. Since Cai et al. (2009) suggested that REM sleep, which occurs to a greater degree in the latter half of a night’s sleep, increases spreading activation, the performance differences between sleep and wake groups may be more significant with overnight sleep than with a short nap. Furthermore, Payne et al. (2009) found a negative correlation between veridical recall and slow wave sleep (SWS), indicating reduced veridical recall performance with increasing SWS, again suggesting that differences between the sleep and wake groups may be more significant after a longer period of sleep than after a daytime nap.

In this present meta-analysis, we therefore aimed to analyze what effect sleep has on both accurate and false memory in DRM tests. We included six potential moderator variables, and analyzed the possible effect that these may have as constraints on effects of sleep on memory consolidation: (1) whether the memory task is recall or recognition testing, (2) the number of words in each list, (3) the number of different lists learned, (4) whether words were presented auditorily or visually, (5) emotionality of the lists, and (6) whether the study was an overnight or nap study.

Analyzing sleep effects on old, new, and lure words individually is useful for formulating comparisons between recall and recognition tests. However, in recognition tests, any observed changes in accuracy as a consequence of sleep could be due to changes in discriminability between word types or changes in response biases to respond yes more or less often. We therefore also used signal detection measures to distinguish the overall sensitivity or discriminability (*d'*) and response bias (*C*) between sleep and wake groups for the studies testing recognition memory. We distinguished true recognition, defined as differences in responses to old words and new (unrelated) words, and false recognition, defined as differences in responses to lure words and new words. We hypothesized that sleep groups would have larger discriminability and response bias scores than wake groups for true recognition, which would indicate that the sleep groups are more likely to correctly accept old words as previously seen and to accurately reject new words as unseen. If so, this would provide evidence in support of a positive role of sleep on memory consolidation and improving accuracy of memory. In contrast, the effects of sleep on false recognition are still under review, and so we might expect to see a larger discriminability and response bias score for the sleep groups if sleep increases false recognition (Monaghan et al., 2017), larger scores for the wake groups if sleep reduces false recognition (Fenn et al., 2009), or no difference in discriminability and response bias if sleep does not influence false recognition (Diekelmann et al., 2008).

## Method

To collect the relevant data, we conducted searches in both Scopus and Web of Science [23-06-2017], using the keywords “sleep OR nap AND false memories.” Scopus produced 113 results, and Web of Science produced 139 results. Our next step was then to check for duplicates, yielding a total of 169 unique entries. An additional two articles from our own research lab were also included in the final analysis, although these were not produced during the main searches due to being submitted for review or in preparation at the time of the searches. These entries were then screened using the following inclusion criteria: (1) Behavioral studies conducted with adult participants, who were (2) exposed to DRM word lists and (3) asked to take part in a recall or recognition task (4) after a period of sleep (which could be overnight or a nap), with (5) a wake group comparison condition. This screening led to the inclusion of nine articles in total, with some of those containing multiple experiments (13 individual experiments with a total of 596 participants overall; see Table 1 for summary data and the moderators for each experiment).

**Table 1:** Descriptions of means, standard deviations, Hedge’s *g*, and standard errors for lure, old, and new words for each experiment, as well as information on moderators

	Year	Lure Words				Old Words				New Words				Test Type	No. of Lists	Words in List	Presentation Type	Emotionality	Sleep Type
Authors		Sleep Mean (*SD*)	Wake Mean (*SD*)	Hedge’s *g*	*SE*	Sleep Mean (*SD*)	Wake Mean (*SD*)	Hedge’s *g*	*SE*	Sleep Mean (*SD*)	Wake Mean (*SD*)	Hedge’s *g*	*SE*						
Payne et al. (Exp. 1)	2009	3.600 (1.649)	2.900 (1.697)	0.42	0.03	21.900 (9.895)	15.700 (7.637)	0.70	0.03	5.600 (7.422)	6.200 (5.940)	– 0.09	0.03	recall	8	12	auditory	neutral	overnight
Payne et al. (Exp. 3)	2009	4.300 (1.600)	2.900 (1.497)	0.88	0.14	26.600 (12.000)	26.400 (15.715)	0.01	0.13	8.090 (5.200)	8.600 (9.354)	– 0.08	0.13	recall	8	12	auditory	neutral	nap
Diekelmann et al. (low performers)	2010	2.860 (1.323)	1.000 (1.323)	1.32	0.31	25.140 (24.780)	21.430 (9.366)	0.19	0.25	4.710 (2.884)	4.430 (2.884)	0.09	0.25	recall	8	12	auditory	neutral	overnight
Diekelmann et al. (high performers)	2010	1.640 (1.194)	2.270 (1.194)	– 0.51	0.17	43.730 (31.130)	50.360 (9.386)	– 0.28	0.17	2.820 (2.885)	4.180 (2.885)	– 0.45	0.17	recall	8	12	auditory	neutral	overnight
McKeon et al.	2012	2.267 (1.579)	0.800 (0.862)	1.12	0.15	14.067 (5.675)	7.600 (4.733)	1.20	0.15	2.133 (1.685)	2.667 (2.845)	– 0.22	0.13	recall	10	10	auditory	emotional	overnight
Fenn et al. (Exp. 1)	2009	0.690 (0.203)	0.760 (0.203)	– 0.34	0.04	0.560 (0.920)	0.590 (0.136)	– 0.05	0.04	0.180 (0.136)	0.220 (0.203)	– 0.23	0.04	recognition	16	15	auditory	neutral	overnight
Fenn et al. (Exp. 2)	2009	0.740 (0.120)	0.860 (0.120)	– 0.97	0.13	0.720 (0.640)	0.710 (0.120)	0.02	0.12	0.150 (0.160)	0.190 (0.160)	– 0.24	0.12	recognition	10	15	visualy	neutral	overnight
Fenn et al. (Exp. 3)	2009	0.630 (0.170)	0.730 (0.167)	– 0.59	0.06	0.570 (0.640)	0.620 (0.167)	– 0.10	0.06	0.210 (0.113)	0.310 (0.167)	– 0.69	0.07	recognition	10	15	visual	neutral	overnight
Diekelmann et al. (Exp. 1)	2008	0.770 (0.116)	0.750 (0.112)	0.17	0.13	0.670 (0.300)	0.700 (0.112)	– 0.13	0.13	0.270 (0.116)	0.280 (0.112)	– 0.09	0.13	recognition	18	15	auditory	neutral	overnight
Lo et al.	2014	0.730 (0.187)	0.870 (0.112)	– 0.88	0.15	0.740 (0.150)	0.780 (0.075)	– 0.33	0.14	0.510 (0.224)	0.520 (0.224)	– 0.04	0.13	recognition	10	15	auditory	neutral	overnight
Monaghan et al.	2017	0.726 (0.159)	0.574 (0.153)	0.96	0.08	0.668 (0.167)	0.606 (0.134)	0.40	0.07	0.221 (0.170)	0.215 (0.178)	0.04	0.07	recognition	12	10	visual	neutral	overnight
Newbury & Monaghan	2018	0.587 (0.125)	0.625 (0.178)	– 0.25	0.07	0.520 (0.132)	0.561 (0.158)	– 0.28	0.07	0.237 (0.160)	0.222 (0.167)	0.09	0.07	recognition	15	12	visual	emotional	overnight
Shaw & Monaghan	2017	0.740 (0.172)	0.620 (0.172)	0.68	0.13	0.649 (0.124)	0.568 (0.124)	0.64	0.13	0.177 (0.204)	0.271 (0.204)	– 0.45	0.12	recognition	12	10	visual	neutral	nap

### Meta-analysis

The effect sizes reported are the standardized mean difference in proportion of responses to each word type given as old (in the recognition tests) and proportion recalled (in the recall tasks) between the sleep and wake group, with positive values meaning an increased proportion of responses in the sleep as compared to the wake group. Effect sizes were calculated for previously seen (old) words, unseen related (lure) words, and unseen unrelated (new) words, and analyzed separately (see Table 1 for the means and effect sizes). True recognition and false recognition *d'* and C sensitivity measures were also computed for the studies testing recognition memory. When not enough data was provided in the article to calculate effect size and sensitivity measures, authors were contacted for means and standard deviations.

We computed Hedge’s *g* on the basis of the means and variance reported in each study for the wake and sleep groups. Hedge’s *g* is a variation of Cohen’s *d* that corrects for biases due to small sample sizes. We then fitted a random effects model using the R package metafor (Viechtbauer, 2010). A random effects analysis was chosen because this method, in contrast to a fixed effects meta-analysis, allows for inconsistencies between the studies analyzed, calculation of possible sampling error, and assessment of the effects of moderators on the size of the effect (Borenstein, Hedges, Higgins, & Rothstein, 2010). We introduced the six moderator variables, (1) recall or recognition testing, (2) number of words in each list, (3) number of lists learned, (4) whether words were presented auditorily or visually, (5) emotionality of lists, and (6) overnight or nap study to the model, to examine any possible influence of these moderators on the effect size of sleep.

## Results

### Lure words

**Fig. 1:**
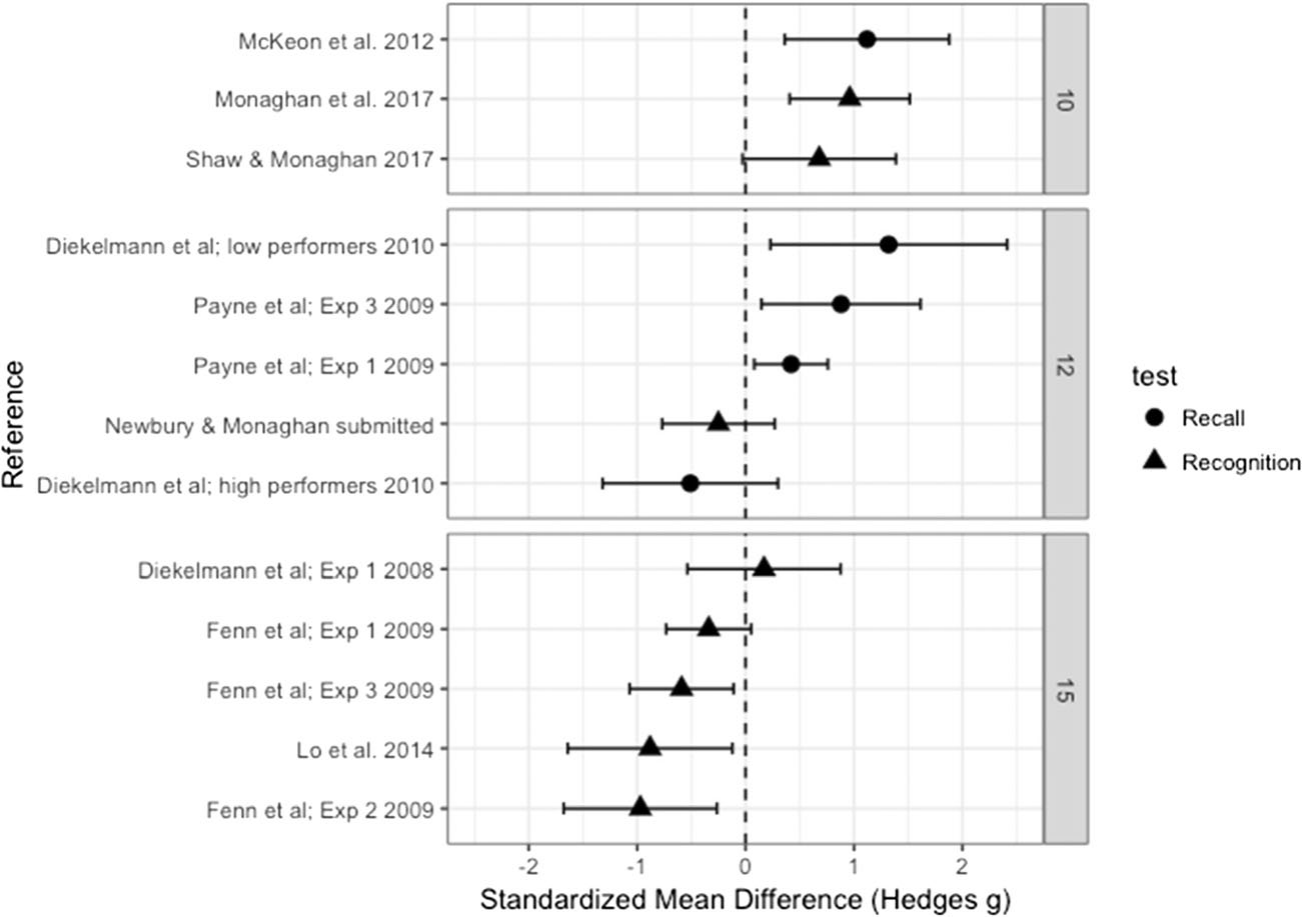
Forest plot containing effect sizes and 95% confidence intervals for the difference in proportions of “old” responses between the sleep and wake groups for lure words. Studies are split by two moderators: number of words in the DRM lists, and recall versus recognition studies. Effect sizes farther to the right indicate more lure words falsely recalled or recognized as old in the sleep than in the wake group, and therefore increased false memories after sleep

The overall effect size for the mean difference in the proportions of responses to lure words given as old between the sleep and wake groups, measured by Hedge’s *g*, was .129 (*SE* = .210), which indicated no significant difference from zero (95% CI [– 0.282, 0.539], *p* = .540). See Fig. 1 for a forest plot of the effect sizes. Since at the time of our data analysis one study within the meta-analysis was unpublished (Newbury & Monaghan, 2018), we conducted a second analysis without this dataset. The overall effect size did not change significantly (Hedge’s *g* = .165, *SE* = 0.227, 95% CI [– 0.281, 0.610], *p* = .469), so we continued our analysis of the full dataset. Possible moderator variables may have led to differing directions of the effects, highlighted by significant heterogeneity [*Q*(12) = 63.227, *p* < .001], indicating that some variance in the data could not be explained by the random measurement error. We therefore analyzed the effect of each of the moderators (see Table 2 for the significance of each moderator).

**Table 2:** Effect of each moderator on the overall effect size difference between sleep and wake groups for lure words

Moderator	*df*	Heterogeneity (*Q*)	*p*
Recall vs. Recognition	1	3.685	.055^+^
Number of lists	1	0.291	.590
Number of words in each list	2	18.368	<.001^***^
Auditory vs. Visual	1	0.387	.534
Emotional vs. Neutral	1	0.264	.608
Nap vs. Overnight sleep	1	1.818	.178

^+^*p* < .1, ^***^p < .001

### Moderator analysis: Recall versus recognition

We found no significant effect of test type [*Q*(1) = 3.86, *p* = .055]. However, since the moderator test was close to significance, we ran effect size analyses of the recall and recognition studies separately. The recall studies showed a medium effect of sleep, with sleep increasing the number of lure words that were falsely recalled as old words, Hedge’s *g* = 0.606 (*SE* = 0.299), which was significantly different from zero (95% CI [0.020, 1.192], *p* = .043). The recognition studies showed a very small, nonsignificant effect in the opposite direction, with sleep reducing the proportion of “old” responses to lure words, Hedge’s *g* = – 0.150 (*SE* = 0.243), indicating no significant difference from zero (95% CI [– 0.626, 0.327], *p* = .538).

### Moderator analysis: Number of words

The studies varied in use of either 10, 12, or 15 words within each list. The moderator test indicated a significant effect of number of words [*Q*(2) = 18.368, *p* < .001]. Studies that used 10 words in each list showed a significant increase in the proportion of lure words falsely recalled or recognized as old after sleep than after being awake (Hedge’s *g* = 0.920, *SE* = 0.193, 95% CI [0.541, 1.300], *p* < .001). No significant effect for 12 words was found (Hedge’s *g* = 0.315, *SE* = 0.302, 95% CI [– 0.277, 0.908], *p* = .297). The effect for 15 words, however, was found to be significantly different from zero (Hedge’s *g* = – 0.495, *SE* = 0.165, 95% CI [– 0.818, – 0.172], *p* = .003), with an *increase* in false memories in the wake as compared to the sleep group.

Since the moderator results also indicated a marginally significant difference in performance between recall and recognition studies, we analyzed whether the significant effect of number of words in each list was evident in only those studies using recognition testing. This effect was confirmed [*Q*(2) = 22.043, *p* < .001], with lists of 10 words leading to increased false recognition after sleep as compared to being awake (Hedge’s *g* = 0.853, *SE* = 0.223, 95% CI [0.417, 1.290], *p* < .001). Lists with 12 words showed no effect (Hedge’s *g* = – 0.250, *SE* = 0.265, 95% CI [– 0.769, 0.269], *p* = .345), whereas word lists with 15 words led to an increase in false recognition in the wake relative to the sleep group (Hedge’s *g* = – 0.495, *SE* = 0.165, 95% CI [– 0.818, – 0.172], *p* = .003). There was insufficient variation in the list lengths in the recall-test studies to allow us to analyze these separately.

### Publication bias

**Fig. 2:**
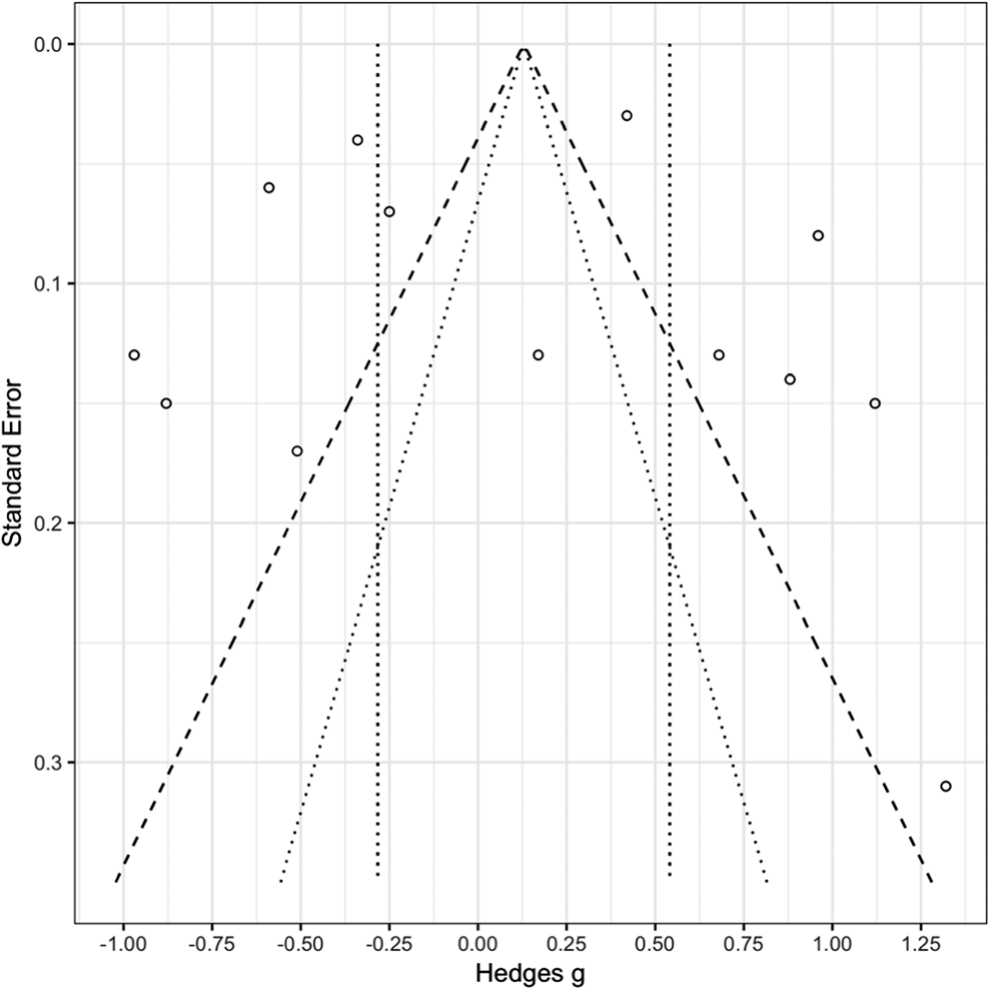
Funnel plot showing the standard errors of effect sizes between sleep and wake groups for lure words, with 95% (dotted lines) and 99% (dashed lines) confidence intervals

Funnel plots show the distribution of effect sizes around the mean based on the sample size, with confidence intervals indicating where studies are likely to be positioned, if there is no publication bias. If many studies fall outside the confidence intervals, this indicates that there may be a publication bias (i.e., only studies with larger effect sizes are published). Figure 2 shows a funnel plot of effect sizes for proportions of lure words given as old in the sleep versus the wake group. An Egger’s regression test for funnel plot asymmetry, used for smaller meta-analyses (< 25 studies), was run to test for possible publication bias (Egger, Smith, Schneider, & Minder, 1997). A number of effect sizes are outside the expected distribution; however, Egger’s regression test indicated no significant funnel plot asymmetry (*z* = 0.910, *p* = .365), and thus no evidence for publication bias.

### Old words

**Fig. 3:**
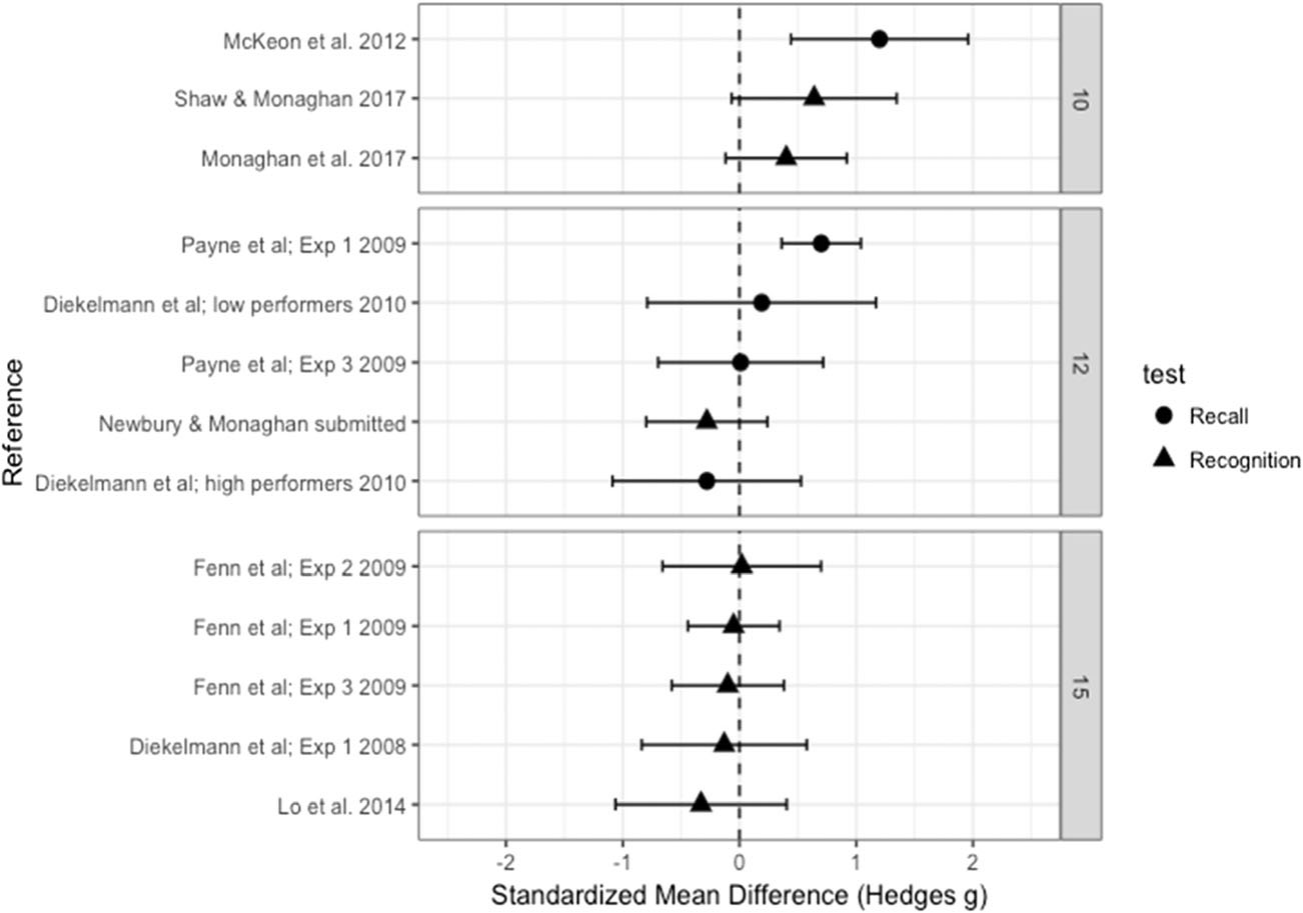
Forest plot containing effect sizes and 95% confidence intervals for the difference in proportions of old words correctly recalled or recognized between sleep and wake groups. Effect sizes farther to the right indicate an increase in the proportion of old words accurately recalled or recognized in the sleep as compared to the wake group

The overall Hedge’s *g* effect size for old words was 0.159 (*SE* = 0.126), which again indicated no significant difference from zero (95% CI [– 0.088, 0.406], *p* = .206); see Fig. 3 for a forest plot of the effect sizes. Again we ran the analysis without the unpublished data, and found no significant change in the effect size (Hedge’s *g* = 0.203, *SE* = 0.130, 95% CI [– 0.052, 0.458], *p* = .458). We therefore continued with the full dataset. There was significant heterogeneity, indicating variance in the data that could not be explained by random measurement error [*Q*(12) = 28.159, *p* = .005]. We therefore again analyzed the effect of each of the moderators (see Table 3 for the significance of each moderator).

**Table 3:** Effect of each moderator on the overall effect size difference between sleep and wake groups for old words

Moderator	*df*	Heterogeneity (*Q*)	*p*
Recall vs. Recognition	1	3.933	.047^*^
Number of lists	1	1.376	.241
Number of words in each list	2	7.151	.028^*^
Auditory vs. Visual	1	0.088	.767
Emotional vs. Neutral	1	0.321	.571
Nap vs. Overnight sleep	1	0.259	.611

^*^p < .05

### Moderator analysis: Recall versus recognition

Recall versus recognition as a moderator had a significant effect [*Q*(1) = 3.933, *p* = .047]. We therefore ran effect size analyses for the recall and recognition studies separately. For studies using a test of recall, we found no significant effect of sleep versus being awake (Hedge’s *g* = 0.407, *SE* = 0.256, 95% CI [– 0.094, 0.909], *p* = .112), nor was there a significant effect for recognition studies (Hedge’s *g* = 0.005, *SE* = 0.100, 95% CI [– 0.190, 0.200], *p* = .958). Therefore, although recall studies differed significantly from recognition studies, with recall studies showing increased performance accuracy after sleep as compared to recognition studies, there was no significant difference in performance accuracy between sleep and wake groups for the tests of recall or recognition analyzed separately.

### Moderator analysis: Number of words

We found the number of words in each list (10, 12, 15) to be a significant moderating variable [*Q*(2) = 7.151, *p* = .028]. We found a medium effect based on 10 words in the lists (Hedge’s *g* = 0.683, *SE* = 0.230, 95% CI [0.231, 1.134], *p* = .003), with an increase in performance accuracy after sleep as compared to being awake. We found no significant effect based on either 12 words (Hedge’s *g* = 0.116, *SE* = 0.505, 95% CI [– 0.334, 0.565], *p* = .614) or 15 words (Hedge’s *g* = – 0.094, *SE* = 0.124, 95% CI [– 0.338, 0.149], *p* = .448).

Again we tested the effect of number of words for recognition studies only. The same significant effect was found [*Q*(2) = 6.841, *p* = .033], with lists of 10 words leading to a significant increase in performance accuracy after sleep relative to being awake (Hedge’s *g* = 0.484, *SE* = 0.213, 95% CI [0.066, 0.902], *p* = .023), and no effect based on either 12 words (Hedge’s *g* = – 0.280, *SE* = 0.265, 95% CI [– 0.797, 0.239], *p* = .290) or 15 words (Hedge’s *g* = – 0.094, *SE* = 0.124, 95% CI [– 0.338, 0.149], *p* = .448).

### Publication bias

**Fig. 4:**
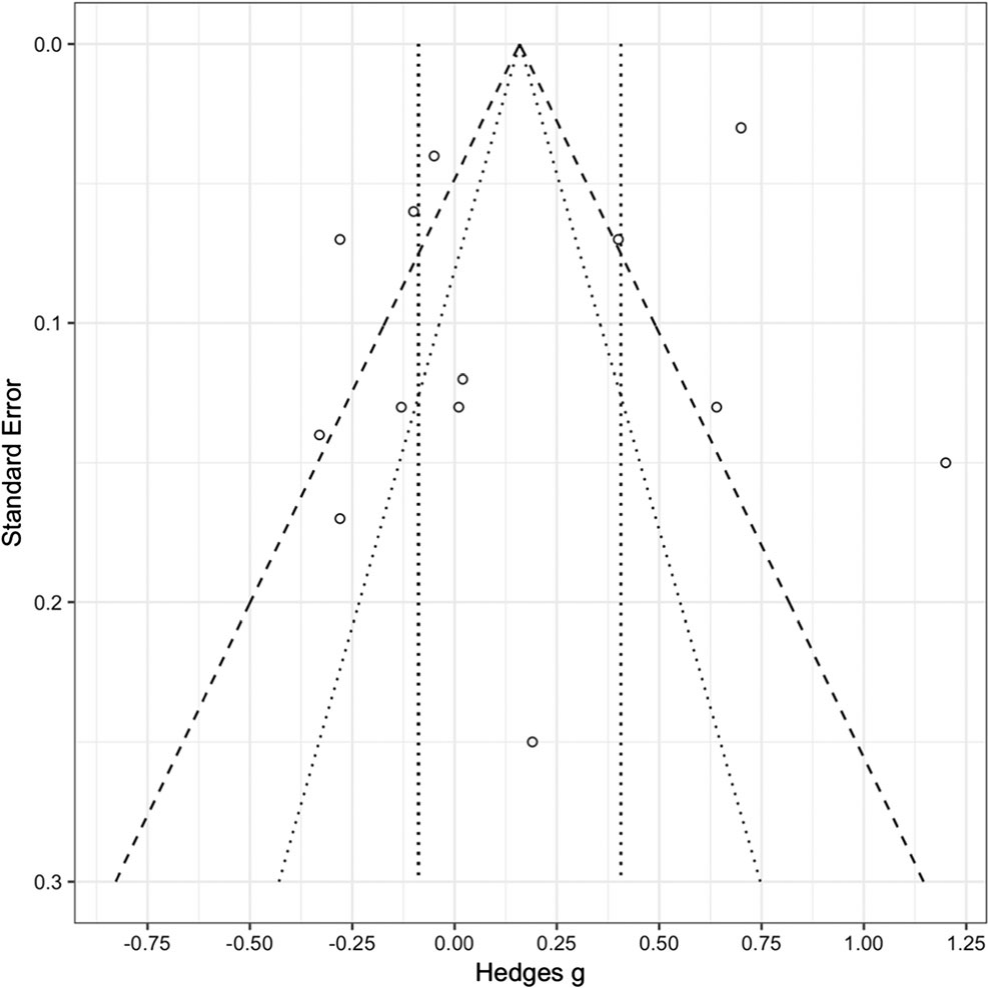
Funnel plot showing the standard errors of effect sizes between sleep and wake groups for old words, with 95% (dotted lines) and 99% (dashed lines) confidence intervals

Figure 4 shows a funnel plot of effect sizes for accurate recall or recognition of previously seen (old) words in the sleep versus the wake group. A number of effect sizes are outside the expected distribution; however, an Egger’s regression test indicated no significant funnel plot asymmetry (*z* = – 0.272, *p* = .786), and thus no evidence of publication bias.

#### New words

**Fig. 5:**
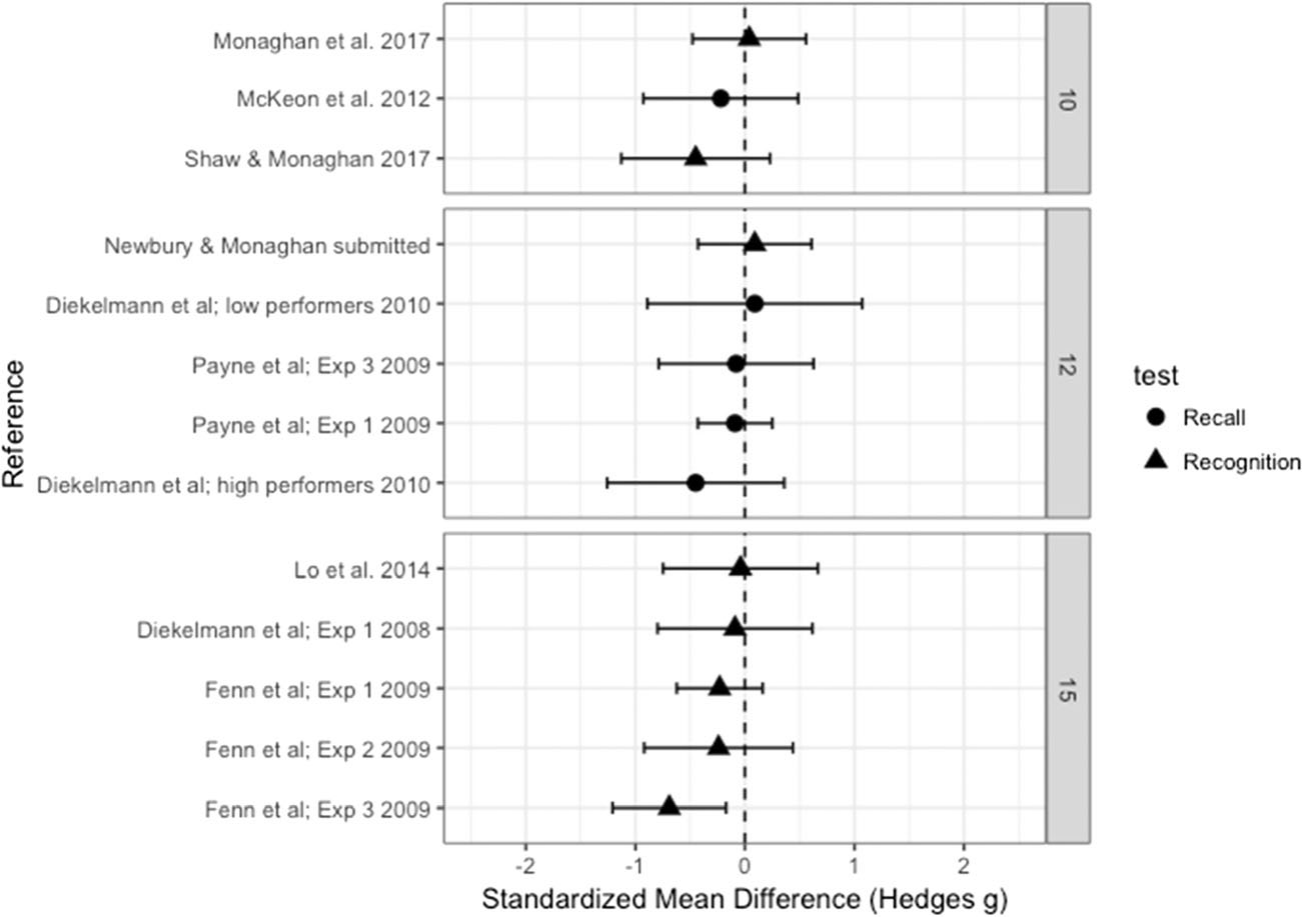
Forest plot containing effect sizes and 95% confidence intervals for the difference in proportions of new words recalled or recognized as “old” between sleep and wake groups. Effect sizes farther to the right indicate an increase in the proportion of new words falsely recalled or recognized as “old” in the sleep as compared to the wake group

For new words, the overall Hedge’s *g* effect size was – 0.277 (*SE* = 0.079), which significantly differed from zero (95% CI [– 0.333, – 0.022], *p* = .026), suggesting that new words were falsely recalled or recognized as old significantly more in the wake than in the sleep group; see Fig. 5 for a forest plot of the effect sizes per experiment. Removing the unpublished data did not significantly change the results (Hedge’s *g* = – 0.204, *SE* = 0.083, 95% CI [– 0.367, – 0.041], *p* = .014), so we continued with the full dataset. Unlike for lure and old words, heterogeneity was not significant, suggesting that moderators were not influencing the effect and that any variance in the data can be explained by random measurement error [*Q*(12) = 7.440, *p* = .827].

### Publication bias

**Fig. 6:**
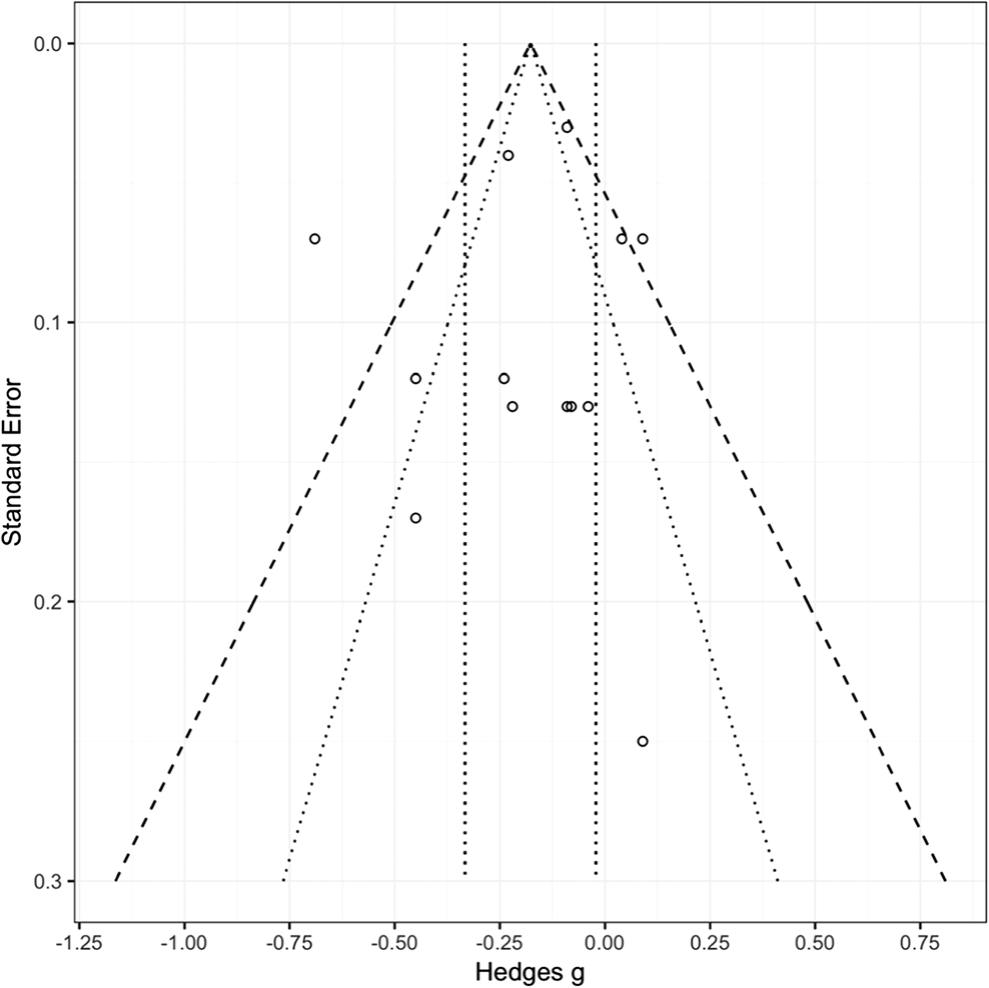
Funnel plot showing the standard errors of effect sizes between sleep and wake groups for new words, with 95% (dotted lines) and 99% (dashed lines) confidence intervals

Figure 6 shows a funnel plot of effect sizes for accurate rejection of new words not previously seen for the sleep versus wake groups. Only two effect sizes are outside the expected distribution; an Egger’s regression test indicated no significant funnel plot asymmetry (*z* = – 0.179, *p* = .858), and thus no evidence of publication bias.

#### Signal detection analyses

For those studies in which participants were given a recognition task, we calculated the mean difference between sleep and wake groups in their overall discriminability (*d'*) and response bias (*C*) for old versus new words (true recognition) and for lure versus new words (false recognition). See Table 4 for the *d'* and *C* scores per experiment.

**Table 4:** Descriptions of discriminability (*d'*) and response bias (*C*) for false recognition (lure vs. new words) and true recognition (old vs. new words) for the sleep and wake groups

	Discriminability (*d'*)				Response Bias (*C*)			
	Sleep Groups		Wake Groups		Sleep Groups		Wake Groups	
Authors	False Recog.	True Recog.	False Recog.	True Recog.	False Recog.	True Recog.	False Recog.	True Recog.
Fenn et al. (Exp. 1)	0.819	0.267	0.794	0.216	2.125	2.401	2.067	2.356
Fenn et al. (Exp. 2)	0.986	0.455	0.980	0.362	1.940	2.205	1.856	2.164
Fenn et al. (Exp. 3)	0.927	0.382	0.860	0.274	1.994	2.266	1.884	2.177
Diekelmann et al. (Exp. 1)	0.713	0.188	0.688	0.192	2.076	2.338	2.075	2.323
Lo et al.	0.498	– 0.014	0.584	0.000	1.703	1.959	1.651	1.943
Monaghan et al.	0.536	0.174	0.433	0.143	1.819	2.000	1.882	2.027
Newbury & Monaghan	0.389	0.038	0.444	0.094	1.956	2.131	1.954	2.129
Shaw & Monaghan	0.636	0.251	0.374	0.020	1.859	2.051	1.816	1.993

### Discriminability (d')

#### False recognition

D-prime (*d'*) for false recognition was analyzed by calculating the *z*-inverse hit rate (lure words falsely accepted as old/total number of lure words) minus the *z*-inverse false alarm rate (new words falsely accepted as old/total number of new words) for each experiment.

**Fig. 7:**
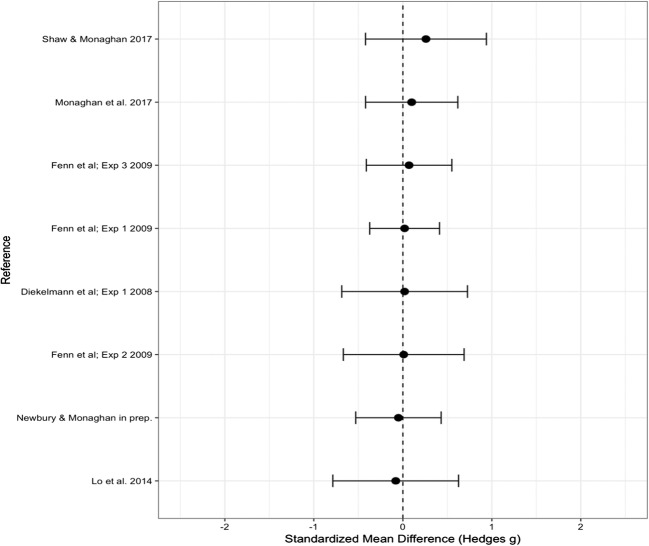
Forest plot containing effect sizes and 95% confidence intervals for false recognition discriminability (*d'*) scores. Effect sizes farther to the right indicate an increase in discriminability for the sleep as compared to the wake group

The overall Hedge’s *g* effect size did not significantly differ from zero (Hedge’s *g* = 0.039, *SE* = 0.098, 95% CI [– 0.153, 0.230], *p* = .692) (see Fig. 7 for the effect sizes). The test of heterogeneity was not significant, suggesting that no potential moderators were influencing the results [*Q*(7) = 0.736, *p* = .998].

#### True recognition

D-prime (*d'*) for true recognition was analyzed by calculating the *z*-inverse hit rate (number of hits/total number of old words) minus the *z*-inverse false alarm rate (new words falsely accepted as old/total number of new words) for each experiment.

**Fig. 8:**
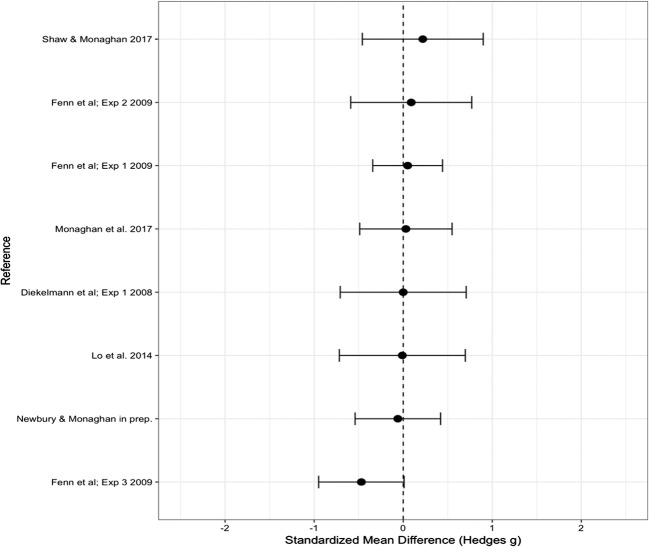
Forest plot containing effect sizes and 95% confidence intervals for true recognition discriminability (*d'*) scores. Effect sizes farther to the right indicate an increase in discriminability for the sleep as compared to the wake group

The overall Hedge’s *g* = – 0.044 (*SE* = 0.098), which did not significantly differ from zero (95% CI [– 0.236, 0.147], *p* = .650) (see Fig. 8). The test of heterogeneity was not significant, suggesting that no potential moderators were influencing the results [*Q*(7) = 4.082, *p* = .770].

### Response bias (C)

#### False recognition

**Fig. 9:**
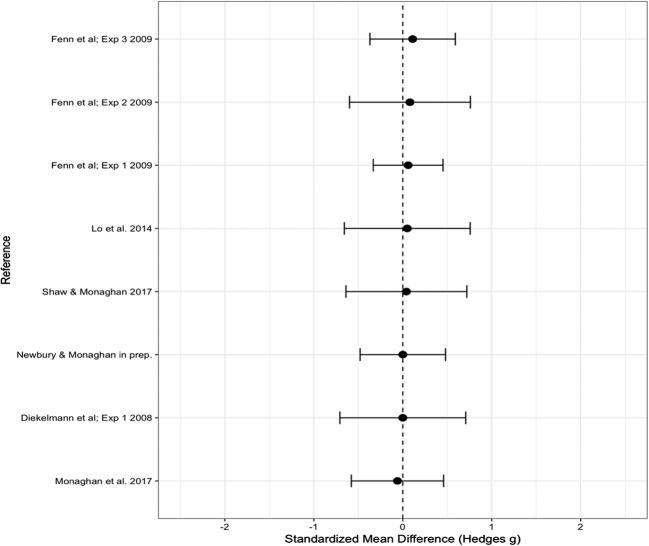
Forest plot containing effect sizes and 95% confidence intervals for false recognition response bias (*C*) scores. Effect sizes farther to the right indicate more conservative responses in the sleep than in the wake group

Response bias (*C*) for false recognition was calculated by the *z*-inverse transformation of [hit rate (lure words) + false alarm rate]/2. We found no significant effect of sleep on response bias (Hedge’s *g* = 0.037, *SE* = 0.098, 95% CI [– 0.155, 0.229], *p* = .706); see Fig. 9 for the effect sizes. There was no significant heterogeneity, indicating that no potential moderators were influencing the effect [*Q*(7) = 0.287, *p* = 1.000].

#### True recognition

**Fig. 10:**
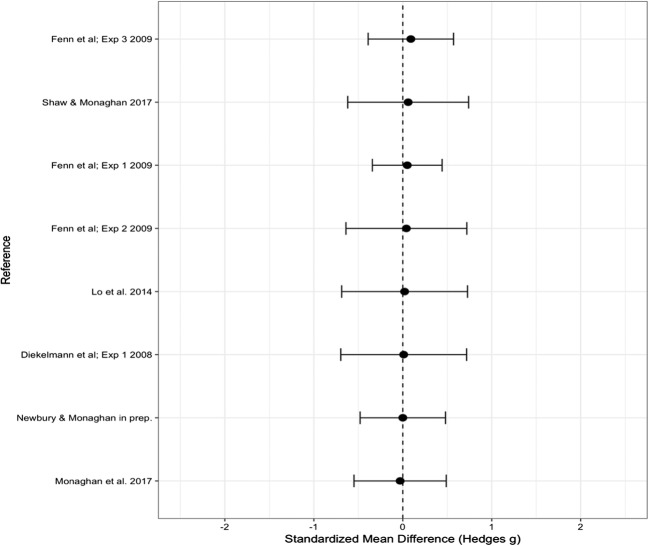
Forest plot containing effect sizes and 95% confidence intervals for true recognition response bias (*C*) scores. Effect sizes farther to the right indicate more conservative responses in the sleep than in the wake group

Response bias (*C*) for true recognition was calculated by the *z*-inverse transformation of [hit rate (old words) + false alarm rate]/2. We found no significant effect of sleep on response bias for true recognition (Hedge’s *g* = 0.032, *SE* = 0.098, 95% CI [– 0.159, 0.224], *p* = .741); see Fig. 10. There was no significant heterogeneity, indicating that no potential moderators were influencing the effect [*Q*(7) = 0.148, *p* = 1.000].

## Discussion

The present study examined the effect of sleep on consolidation of seen words, as well as susceptibility to false memories using the DRM procedure. Although we found no overall significant effect of sleep on false memories, the present meta-analysis helps to clarify mixed findings within the literature, by demonstrating that recall versus recognition testing, and shorter list lengths, enhance sleep-based increases in DRM false memories.

Based on the conclusions of a previous meta-analysis conducted by Chatburn et al. (2014), we hypothesized that this lack of an overall effect may have been due to differences between tests of recall and recognition. On the basis of the previous meta-analysis, as well as the studies presented in the present analysis, we predicted a strong enhancement effect of sleep relative to being awake on false recall (Payne et al., 2009). In contrast, for tests of false recognition, sleep has been found to reduce, have no effect, or enhance false memories (Diekelmann et al., 2008; Fenn et al., 2009; Monaghan et al., 2017). A moderator test examining the effect of sleep on false recall and recognition separately found a significant effect of recall, with greater false memories after sleep than after being awake, whereas recognition tests did not have this same effect. The lack of an effect of sleep on false recognition was further supported by the signal detection analysis, which revealed no significant difference in discriminability or response bias between sleep and wake groups. Thus, this meta-analysis supports Chatburn et al.’s (2014) smaller meta-analysis indicating a significant effect of sleep on false recall, but no effect for false recognition.

However, the larger set of studies investigated in the present meta-analysis enabled us to go further to determine the role of additional task constraints on the effect of sleep on memory. In particular, the results also indicated that list length moderated the effect of sleep on false memories. The studies examined in this analysis used lists consisting of 10, 12, or 15 words. Based on previous research indicating an increase in false memories when more list items were presented, due to increasing associations (Robinson & Roediger, 1997), we predicted two possible hypotheses. If sleep aids in spreading activation of memories equally regardless of the density of the word lists, then we would expect to see no effect of list length on the overall effect size. However, word lists of shorter list length create fewer semantic associations at encoding, thus priming fewer similar, unseen words. If sleep aids memory by increasing the spreading activation in long-term semantic associative memory, then this is more likely to result in the activation of lure words for shorter lists, where the activation within a network containing fewer semantically related items is sparse, than for the more densely activated networks resulting from a longer list (Cai et al., 2009; Sio et al., 2013). The results of the analysis supported this, with an increase in false memories after sleep when studies used lists of 10 words, whereas studies containing lists of 15 words led to a reduction in the proportion of “old” responses to lure words in the sleep as compared to the wake group.

For old words, we also found no overall significant difference between the sleep and wake groups on memory performance. This contrasts with previous literature examining the positive effect of sleep on veridical memory consolidation (Dumay & Gaskell, 2007; Plihal & Born, 1997; Wilson et al., 2012). Despite this, two moderators were found to influence the effect size. Tests of recall led to greater performance accuracy after sleep than did tests of recognition for veridical memory of old words. We also found an effect of list length; shorter word lists of ten words led to an increase in accurate memory performance after sleep than after being awake. Therefore, sleep appears to be more beneficial when participants were required to encode fewer words per list. Importantly, this enhancement of sleep effects from short lists for both false and veridical memory was not due to an increase in response bias associated with sleep, as confirmed by the signal detection analyses. The effects were rather specific: Only for sparse sets of thematically related words did sleep improve recognition of old words, and increase acceptance of related but unseen lure words.

For unseen, unrelated (new) words, we expected to see either no difference in performance between sleep and wake groups, due to higher performance accuracy evident in both groups (McKeon, Pace-Schott & Spencer, 2012; Monaghan et al., 2017; Newbury & Monaghan, 2018), or an increase in accurate rejection of new words after sleep than after being awake due to an overall increase in performance accuracy after sleep (Rasch & Born, 2007). The meta-analysis revealed a small increase in the proportion of new words falsely recalled or recognized as old in the wake group than in the sleep group. Therefore, the sleep groups were significantly more accurate at rejecting new words as previously seen, supporting previous research indicating a benefit of sleep on accurate memory performance (Davis, Di Betta, Macdonald, & Gaskell, 2009; Dumay & Gaskell, 2007; Plihal & Born, 1997; Wilson et al., 2012).

Although the present results cannot be extended to apply to general verbal memory consolidation, as the DRM paradigm is designed primarily to examine susceptibility to DRM false memories, and not to investigate veridical memory performance, it should be noted that veridical and false memory within DRM tests are often correlated (e.*g*., Payne et al., 2009; Shaw & Monaghan, 2017). For those studies that used recall testing and shorter word lists, we saw both an increase in veridical memory, and greater susceptibility to false memories after sleep than after being awake. This, along with the finding that unseen, unrelated new words were rejected more easily by the sleep group, provides support for spreading activation theories of sleep and memory. The present results indicate a role of sleep in associative activation theory (Howe et al., 2009; Roediger et al., 2001), suggesting that shorter word lists with fewer semantic associations benefit from sleep-dependent spreading activation, leading to false acceptance of critical lures to a greater extent than after being awake, as well as accurate rejection of words with no sematic association.

### Conclusions

The present meta-analysis of the effects of sleep on veridical and false memory consolidation in DRM tests indicated no overall significant effects. Despite this, it is clear that several moderating variables influence offline memory consolidation. Furthermore, the studies presented in this meta-analysis contain further differences in methodology that may explain the lack of an effect of sleep on both veridical and false memories. For instance, Newbury and Monaghan (2018) found that sleep improved the consolidation of old words to a greater extent than did being awake, but only for word lists of negative valence. Monaghan et al. (2017) and Shaw and Monaghan (2017) found evidence for sleep aiding veridical consolidation specifically for those word lists presented to the left hemisphere. Furthermore, Lo, Sim, and Chee (2014) found a reduction in false recognition specifically in older adults, who have previously been found to show different levels of susceptibility to false memories than do young adults (Dennis, Kim, & Cabeza, 2007; Kensinger & Corkin, 2004), whereas Diekelmann et al. (2010) found an increase in false recall after sleep only for those participants who had overall low general memory performance. The DRM paradigm does however provide us with evidence for only one type of false memory illusion. Thus, we cannot make firm conclusions regarding the effects of sleep on other forms of veridical and false memories, for example during eye witness testimony or autobiographical memory for past events.

In conclusion, sleep may therefore improve performance accuracy differentially depending on a number of factors, but the present results do indicate that observations of sleep enhancement of veridical and false memory effects are task-dependent—potentially sensitive to source monitoring constraints in memory tasks—as well as subject to constraints emergent from the structure of semantic associative memory, as measured by list length, which reflects the density of interconnections within networks of associated words (Monaghan et al., 2017; Robinson & Roediger, 1997). Further investigation as to the effects that these different factors may have on the integration and consolidation of specific information from the short-term to the long-term memory stores will allow for a greater understanding as to the complexities of memory consolidation under different conditions.
